# Cellulosic Textiles—An Appealing Trend for Different Pharmaceutical Applications

**DOI:** 10.3390/pharmaceutics15122738

**Published:** 2023-12-06

**Authors:** Giuseppina Nocca, Alessandro Arcovito, Nermeen A. Elkasabgy, Mona Basha, Noah Giacon, Elena Mazzinelli, Mohammed S. Abdel-Maksoud, Rabab Kamel

**Affiliations:** 1 Dipartimento di Scienze Biotecnologiche di Base, Cliniche Intensivologiche e Perioperatorie, Università Cattolica del Sacro Cuore, Largo Francesco Vito 1, 00168 Rome, Italy; giuseppina.nocca@unicatt.it (G.N.); alessandro.arcovito@unicatt.it (A.A.); elena.mazzinelli@unicatt.it (E.M.); 2 Fondazione Policlinico Universitario “A. Gemelli”, IRCCS, Largo Agostino Gemelli 8, 00168 Rome, Italy; 3 Department of Pharmaceutics and Industrial Pharmacy, Faculty of Pharmacy, Cairo University, Kasr El-Aini Street, Cairo 11562, Egypt; 4 Pharmaceutical Technology Department, National Research Centre, Cairo 12622, Egyptdrrababk@hotmail.com (R.K.); 5 Medicinal & Pharmaceutical Chemistry Department, National Research Centre, Cairo 12622, Egypt

**Keywords:** cellulose, fibers, textiles, regenerative medicine, antimicrobial, antiviral

## Abstract

Cellulose, the most abundant biopolymer in nature, is derived from various sources. The production of pharmaceutical textiles based on cellulose represents a growing sector. In medicated textiles, textile and pharmaceutical sciences are integrated to develop new healthcare approaches aiming to improve patient compliance. Through the possibility of cellulose functionalization, pharmaceutical textiles can broaden the applications of cellulose in the biomedical field. This narrative review aims to illustrate both the methods of extraction and preparation of cellulose fibers, with a particular focus on nanocellulose, and diverse pharmaceutical applications like tissue restoration and antimicrobial, antiviral, and wound healing applications. Additionally, the merging between fabricated cellulosic textiles with drugs, metal nanoparticles, and plant-derived and synthetic materials are also illustrated. Moreover, new emerging technologies and the use of smart medicated textiles (3D and 4D cellulosic textiles) are not far from those within the review scope. In each section, the review outlines some of the limitations in the use of cellulose textiles, indicating scientific research that provides significant contributions to overcome them. This review also points out the faced challenges and possible solutions in a trial to present an overview on all issues related to the use of cellulose for the production of pharmaceutical textiles.

## 1. Introduction

A healthy body is an intrinsic requirement for all human beings. Pharmaceutical textiles, like cellulosic textiles, are widely used in our daily lives for several purposes like wound healing, drug delivery, and tissue regeneration [1,2,3,4].

The production of textiles is a very long and complex process that involves preparation of fibers, as well as numerous raw materials, which is the starting point. This multistage process starts from the producing of fibers and continues till the final fabric is created [5]. Production steps comprise spinning, fabric formation through knitting/weaving, followed by the so-called wet–processing. which includes pretreatment procedures through desizing, scouring, bleaching, and then dyeing and finishing—hence producing the final fabric. These stages differ depending on the nature of the used fibers as well as the properties of the desired products allowing for the production of the required textiles [6].

Fibers are either natural or man-made. Natural fibers represent those of vegetable origin (cotton, hemp, and flax) or from animals (wool and silk). For many years, natural fibers were the prevailing type in the textile market. However, the elevated expenses of using arable areas together with the increasing demand for more fibers resulted in enormous progress in man-made fibers production [7]. Cellulose is one of the main materials used for the fabrication of fibers.

Cellulose, as a natural polymer, has many advantages over synthetic polymers. It is a cheap and eco-friendly polymer in addition to its excellent mechanical properties, such as flexural and tensile strengths, as well as its thermal stability that enhances the usage of cellulose instead of synthetic polymers [8]. It is composed of small units called D-glucose, which can be obtained from different natural sources—mainly plants. A huge amount of cellulose is obtained from agricultural wastes. Based on the physical and chemical characters of cellulose, many structural modifications can be applied to improve its physical properties and reactivity. The modifications can be divided into physical and chemical. Physical modification controls shape and size and leads to the formation of micro- and nano-cellulose, while chemical modification includes oxidation, esterification, etherification, grafting, and hybridization.

The multiple benign properties of cellulose have encouraged its exploitation in the pharmaceutical field [9,10,11,12]. Recently, our team used nanocellulose isolated from agro-wastes (e.g., sugarcane bagasse) as a pharmaceutical ingredient for formulations designed for tissue regeneration [13,14,15,16,17,18,19].

Over the past few years, tissue engineering has become an alternative to tissue or organ transplantation. Tissue engineering is a vital process that involves the use of live tissues, growth factors, and biomaterials for improving or replacing injured tissues. The regeneration procedure totally replaces the damaged tissues through proliferating the adjacent intact cells [20,21]. Nanofibers have been widely investigated for tissue engineering applications due to their ability to produce more complex macro structures than scaffolds and sutures. They also represent a suitable structural replica of the extra cellular matrix (ECM) that is mainly composed of nanofibrous protein, allowing cell attachment, growth, differentiation, and organization [22]. The nanofibrous scaffolds are characterized by their biocompatibility, thus providing the needed substrate for tissue regeneration with complete and safe disintegration.

Also, cellulosic and nanocellulosic pharmaceutical textiles have emerged as a remarkable avenue in the pursuit of innovative solutions for infection control within the healthcare sector. Cellulosic textiles, such as cotton, have paved the way for the integration of antimicrobial formulations, opening up a realm of possibilities for enhanced medical textiles with profound implications. However, since cellulose lacks inherent antimicrobial activity, fibers can be readily loaded with antimicrobial agents. The hydroxyl groups present in cellulose and nanocellulose make this possible. Techniques like oxidation, esterification, and etherification are commonly employed to attach compounds that confer biocidal capabilities. These modifications allow for the incorporation of various antimicrobial agents into cellulose [23,24]. Bacterial infection of the skin can result in serious complications such as microbial resistance and dose-dependent toxicities besides the significant delay of the healing process, especially in complicated conditions as in surgical procedures, traumatic injuries, and burn wounds [25]. Thus, more attention has been directed towards the development of effective antimicrobial dressing. Recently, novel antimicrobial nanofibers have been introduced as antimicrobial dressings with high interconnected porosity (60–90%), gas permeability, balanced moisture, and great absorbance with structures similar to that of the extracellular matrix, thus providing suitable environments capable of protecting injured areas from exogenous infections [26]. However, even nanocellulose is not capable of controlling bacterial infection itself, as it has no antimicrobial activity, but nanofibers can be readily loaded with antimicrobial agents, metal nanoparticles, and natural and synthetic products [23,27,28].

This review article is focused on clarifying cellulose sources and physicochemical properties, and polymorphs are also covered together with the methods of preparation of specific cellulosic fibers for pharmaceutical use. The different methods of chemical modifications are also highlighted. Moreover, different aspects are examined related to cellulose- and nanocellulose-based textiles and their pharmaceutical applications, mainly for tissue engineering and anti-microbial, anti-viral, and wound dressing applications. Merging between cellulose and other materials like drugs, metal nanoparticles, and plant-derived materials as well as synthetic materials are also illustrated. This review also highlights some of the challenges and limitations faced due to the use of cellulose textiles in pharmaceutical fields. Researchers are addressing these challenges with great commitment, as most of the referenced works discussed in this review cover the period from 2020 to 2023. The substantial number of publications in these years emphasizes the tremendous scientific dedication to this topic.

## 2. Origin of Cellulose and Its Physicochemical Properties

Cellulose is one of the most abundant natural compounds. Similarly to other natural products, such as chitosan and gelatin, it is used in different medical applications related to human healthcare [29]. Cellulose is a polymer composed of small units called D-glucose and can be found in different organisms from bacteria and plants to algae and marine animals [30]. The major cellulose production (about 140 billion tons) is of plant origin [31]. It can be used directly as extracted from natural sources or can be chemically modified to enhance its physicochemical properties.

**Figure 1:**
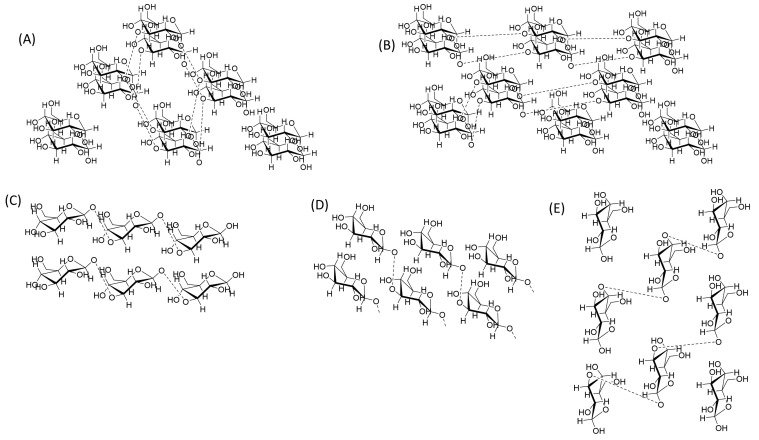
Crystal structures of different cellulose subtypes (**A**) Type Iα, (**B**) Type Iβ, (**C**) type II, (**D**) Type III, and (**E**) Type IV.

Based on the origin and method of preparation, there are four main subtypes of cellulose based on the arrangement of the monomer in the crystal structure. These types are I, II, III, and IV (Figure 1). Cellulose type I can be divided into two main subtypes, Iα and Iβ, and both types can be found in algal cell walls. Subtype Iβ exists in high percentages in higher plant and marine creatures such as Halocynthiaroretzi [32]. Cellulose I is considered a native type of cellulose, and it consists of 15,000 sugar units and decomposes at 400 °C [33]. It is composed of parallel polymer chains with highly random arrangements [34].

On the other hand, cellulose type II is produced mainly by mutant bacteria, such as *Acetobacter xylinum*, in a cold atmosphere [35] and is characterized by anti-parallel polymer chains compared to type I [36]. It is worth mentioning that cellulose type II can be converted to type I via mercerization (using an alkali) or solubilization and recrystallization (regeneration) [37]. The regeneration method results in higher crystallinity, purity, and yield compared to mercerization [38]. Mercerization is a technique where textiles (typically cotton) are treated with a caustic solution (mainly NaOH) or sometimes with a liquid ammonia solution to enhance features such as fiber strength, shrinkage resistance, luster, and dye affinity. The process involves treatment of cellulose with different concentrations of NaOH starting from 10 to 50% at room temperature for a specific period of time (4 h) [39].

On the other hand, cellulose type II is more reactive compared to type I, is considered as one of the most useful types of cellulose, and is used to make cellophane [36].

Cellulose III represents the amorphous form of cellulose. It is produced via the thermal treatment of both cellulose I and cellulose II. It has a high surface area as well as the property of high adsorption due to the fact that its crystalline form is destroyed irreversibly during the manufacturing process, especially in the swelling and washing steps [40]. Finally, cellulose IV is produced from type I and type II via thermal treatment and is known as high-temperature cellulose [41].

Cellulose IV1 and IV2 are prepared from cellulose III1 and III2, correspondingly, via thermal treatment in hot glycerol. It has also been stated that cellulose IV1 and IV2 have unit cells of nearly the same size but with different arrangements of cellulose chains—parallel for cellulose IV1 and antiparallel for cellulose IV2 [42].

## 3. Preparation of Different Cellulose Polymorphs

During earlier decades, there were many attempts to separate cellulose (native cellulose type I). Nowadays, the main sourcing of cellulose type I is through the extraction from different plant sources such as wheat husk, rice husk, maize husk, cotton, etc. In addition, bacteria and algae can participate in total cellulose production [43]. On the other hand, the early work of Badenhuizen and Meyer paved the way for the polymorphic transformation of different types of cellulose [44]. The source of cellulose is mainly agricultural waste, and the extraction process can be divided into three major steps. The first step is pre-hydrolysis, in which the main matrix of the plant is destroyed to reach the cellulose-containing parts. The second step is pulping in which an alkali is used to obtain the cellulose fibers. The last step is bleaching with hydrogen peroxide to obtain pure fibers [45]. These three processes ensure that cellulose is produced free of other plant contents such as lignin and hemicelluloses. It is worth mentioning that the product of these steps is cellulose type Iα.

**Figure 2:**
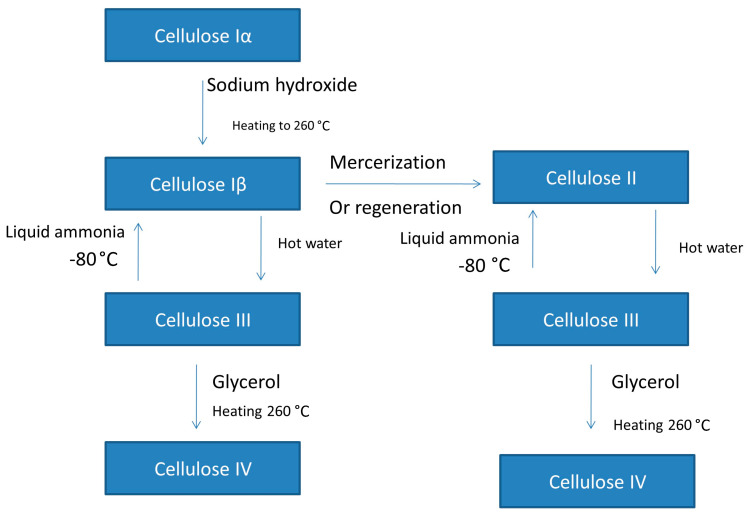
Diagram of the preparation of different cellulose polymorphs.

The production of cellulose polymorphs is summarized in Figure 2. Starting from cellulose type Iα, heating it at a high temperature (260 °C) in the presence of sodium hydroxide will lead to the formation of cellulose type Iβ, which when boiled in water will form cellulose type III. Cellulose type III can be reconverted to cellulose type Iβ using an ammonia solution or another amine at a very low temperature (−80 °C). Also, cellulose type Iβ can be converted to cellulose type II via the mercerization process or regeneration process. Cellulose type II can be converted to cellulose type III using hot water, after which the use of liquid ammonia or an amine at low temperature can regenerate cellulose type II. Cellulose type IV is prepared by heating cellulose type III in glycerol at 260 °C [46].

Although cellulose polymorphs have been extensively researched as multidisciplinary materials, cellulose suffers from a major drawback with the hydrophobic polymeric matrix. The poor fabrication ability and the tendency to clump and aggregate during processing are mainly due to low solubility in organic solvents, as a result of the strong inter- as well as intramolecular bonding [47,48]. At the same time, increasing the temperature does not result in a subsequent increase in solubility, as cellulose is totally insoluble at <300 °C, while it degrades instantly above this temperature [49]. Additionally, the insolubility of cellulose is significantly affected by the length of the polymeric chains as at a polymerization degree higher than that of celloheptaose, the solubility of the cellooligomers reaches zero [50].

This risk of aggregation or clustering, which may occur within fiber walls, due to the collapse of the internal structure during drying is known as hornification. As a result of fiber aggregation, another problem emerges: the consumption of more energy under the high temperatures required to liberate aggregated fibers. However, in serious cases of hornification, highly refined fibers become harder, or sometimes even impossible, to extract during drying. Meanwhile, incomplete drying might result in microbial growth during long periods of storage. Cellulose fibers can aggregate/hornify regardless of the drying method, resulting in aggregates of variable structures and sizes [51,52,53,54].

## 4. Preparation of Specific Cellulose Types for Pharmaceutical Applications

### 4.1. Regenerated Cellulose Fibers (RCFs)

In the beginning of the twentieth century, regenerated cellulose was developed as the first man-made fibers to improve the softness and comfort of woven materials, representing an alternative to natural fibers like silk and cotton. These fibers combine the lustrous and smooth nature of the former together with the remarkable water absorption ability of the latter [55]. In the early twenties, rayon was the first accepted generic name used to label RCF comprising viscose, lyocell, cellulose acetate, modal, and cupro [56]. RCF is considered as the conventional textile raw material utilized in different fields from sportswear to medical and pharmaceutical, either alone or in combination with other fibers [57]. This versatile use of RCF is related to its unique characteristics such as biodegradability, smoothness, moisture absorption, and ease of dyeing in addition to it being soft and comfortable, which identifies it as a skin-friendly material [58,59].

#### 4.1.1. Production of Regenerated Cellulose Fibers (RCFs)

RCFs are typically produced by the dissolution of cellulose, either in its pure form or obtained from plant fibers or wood pulp. Usually, the length of these raw fibers is not suitable for direct textile use, thus they are subjected to further processing [60]. The fibers are dissolved in different solvents producing intermediate polymeric solutions, which are processed using continuous spinning followed by the regeneration of the desired solid fibers [61]. The spinning process is composed of the following main steps: spinning of the preparation, coagulation, drawing, and washing. Firstly, extrusion of the polymeric solution is performed using a syringe pump, spinneret, and coagulation bath. Then, removal of the solvent takes place either using chemical reactions or through diffusion at the coagulation step and the solidification of the solution into polymeric fibers [62]. Drawing is a crucial step in fiber production that enhances molecular alignments, resulting in fibers of better stiffness and strength. This process can be conducted after the extrusion of the polymeric solution from the spinneret during or after drying. Finally, washing is carried out in order to remove any residual impurities [63]. Generally, the most crucial step in RCF production is improving the dissolution of cellulose using both aqueous and non-aqueous solvents. These solvents are classified as non-derivatizing and derivatizing: the former dissolves the polymer via intermolecular interactions, while the latter results in the formation of unstable, ester, or acetal-ether derivatives [64].

##### Conventional Techniques for Production of RCFs

Viscose and lyocell methods are considered to be the most traditionally used industrial techniques for the manufacture of RCF. The viscose process is the most extensively used and was originally explored by Cross and Bevan in 1893, resulting in the production of the first commercial RCF. This method depends on wood pulp as the raw material, while the solvents used are sodium hydroxide and carbon disulfide. However, this method encounters considerable industrial and environmental issues including the limited dissolving capability of the solvents, use of high amounts of chemicals, and release of toxic heavy metals during the dissolution process. In addition to the other obstacles related to uncontrolled side reactions, the use of fossil fuel and energy consumption as well as the high cost of solvent recovery are also issues [65,66].

On the contrary, the lyocell process depends on the direct dissolution of cellulose using a nontoxic and biodegradable solvent—N-methylmorpholine-N-oxide (NMMO). This process is more environmentally friendly due to the significant reduction in the number of consumed solvents as well as the ease of solvent recycling [67]. While unlikely, the high cost of NMMO, the possibility of cellulose degradation as a result of side reactions and byproduct generation, and the elevated temperatures required for the process resulting in the consumption of more energy are considered the main limitations of the lyocell process [68,69].

##### Environmental-Friendly-Based Techniques for Production of RCFs

The need for a green process for the production of RC textile fibers has been increasingly growing in recent years. The main challenge in fiber production is the dissolution of cellulose in a suitable solvent capable of breaking the strong hydrogen bonds between cellulose molecules, which is crucial for RCF spinning [7]. Thus, a number of solvents that can effectively dissolve cellulose molecules with minimum hazards and toxicity have been explored. Alkali/urea solvents have been proposed as alternatives to classical solvents, and these are characterized by their low cost, non-toxicity, and fast dissolution. However, the constructed fibers have poor mechanical properties, and the process is energy-intensive [70].

Most recently, ionic liquids (ILs) have been widely investigated since 2002, gaining much attention as an alternative to NMMO. These are liquids composed of cations and anions, therefore they can be designated as salts in the liquid state [71]. They can dissolve cellulose through hydrogen bonding with cellulose chains allowing for the preparation of cellulosic solutions even at high concentrations of cellulose [72]. ILs can be considered as environmentally friendly solvents with very low flammability, practically no vapor pressure, and high thermal stability besides their distinguished capacity for cellulose dissolution [73,74]. Moreover, ILs allow for the production of RCFs at lower temperatures owing to their wide range of melting points varying from −40 to 400 °C [75]. Furthermore, the features of ILs can be readily altered and modified according to the required application through changing the combination of cations and anions [71]. Unfortunately, the main challenges facing the establishment of the large-scale industrial use of ILs are their high cost in addition to reusability problems [76].

### 4.2. Cellulose Nanofibers (CNFs)

In the few past decades, academics have shown growing interest towards exploring nanotechnology in the production of nanofibers and investigating their newly unique distinguished size-dependent properties [77]. The characteristics of developed nanofibers depend mainly on their diameter, length, fiber shape, either hollow or core-shell, surface area, and texture. Nanofibers have a number of exceptional novel features including their high surface area and promoted mechanical, physical, and organic properties as well as their good permeability and compatibility. Therefore, they are considered as promising candidates for many healthcare and medical applications comprising drug delivery, wound dressing, and tissue engineering [78].

Cellulose nanofibers (CNFs)—which are also known as nanofibrillated cellulose (NFCs), microfibrils, macrofibrillated cellulose, nanocrystalline cellulose, or crystallites [79]—are thin nanosized fibrils with a diameter less than 100 nm and several micrometers in length. Such fibrils are characterized by their dense fibrillary network structure with crystalline and amorphous areas. The amorphous region is responsible for the plasticity and flexibility of the CNF, while their stiffness and elasticity are attributed to the crystalline region [25]. As of recently, the production of CNFs can be performed using different cellulosic resources. Several processing techniques have been developed for CNF fabrication [80,81]. These techniques can be typically categorized into (i) mechanical, (ii) biological, (iii) physical, and (iv) chemical.

#### 4.2.1. Production of CNFs

##### Mechanical Methods

These procedures rely on using a number of mechanical treatments including, e.g., grinding, cryocrushing, homogenizing, and microfluidization. The selection of a specific technique depends on some factors relating to the starting materials—their type, chemical and morphological properties, the extent of fibrillation, and the required specifications as well as the final application of the produced CNFs [82]. In the grinding process, fibrillation is performed by breaking up the interchain hydrogen bonds of cellulose by forcing cellulose suspension between the rotating and static grindstones of an ultrafine grinder. The formed cellulose fibers pass through repeated cyclic stresses that the centrifugal forces produce as a result of the shear stress on the fiber suspension, and repeated cyclic pressure finally results in the fibrillation of nanofibers [83].

Cryocrushing is another method used for the preparation of nanofibers. The cellulosic fibers are first swollen in water and frozen in liquid nitrogen, after which they are subjected to high shear forces. Production of fibers depends on the rupturing of the cell walls as a result of the exerted pressure by the ice crystals, thereby liberating the nanofibrils [84].

The high pressure homogenization technique was first applied in 1983 to produce CNF from wood pulp. In this process, cellulose pulp is pretreated using a diluted cellulose suspension and then pumped under a high pressure of 50–2000 bar through a narrow nozzle using a piston into a container to produce nanocellulosic fibers. The particle size of the fibers is controlled by several factors involving the applied high pressure, number of passages through the homogenizer, velocity, and applied shear forces. Increasing these forces increases the shear rates resulting in size reduction. This procedure is characterized by being simple and highly effective without the use of organic solvents [83]. However, the main drawbacks of this method include clogging of the homogenizer, inadequate disintegration of the fibers, consumption of high energy, and the need for repeated mechanical treatment in order to create nanocellulosic fibers of high-quality [85].

In microfluidization, the same steps are followed with high pressure homogenization in the presence of an intensifier pump to provide more pressure. The microfluidizer produces smaller nanocellulosic fibers compared to high pressure homogenization. However, the larger surface area and high amount of OH groups may lead to the agglomeration of the generated fibers [86].

##### Biological Methods

This process depends upon disrupting the crystalline nature of the cellulose nanofibrils utilizing one of the following treatments: microorganism-assisted hydrolysis (enzymes, bacteria, algae) [87], mild mechanical procedures [88], or a combination of more than one [89]. Enzymatic hydrolysis is an efficient process that is based upon using a monocomponent endoglucanase to enhance defibrillation through the specific hydrolysis of the glucosidic bonds along the cellulose chain, mainly in less crystalline regions [90]. The plant cell wall is composed of some essential components comprising cross-linked polysaccharide networks and glycosylated proteins as well as lignin. Cellulose and hemicellulose can be reinforced by lignin, resembling steel rods fixed tightly in concrete. This reinforcement can be effectively eliminated via pretreatment allowing chemicals, enzymes, or biomaterials to easily be in close contact, thus generating more effective products. The pretreatment technique can increase the directly accessible surface area, thus improving cellulose availability. Therefore, the main purpose of enzymatic pretreatment includes the following: firstly, the separation of the cellulose and the removal of hemicelluloses; secondly, reducing the crystallinity of the cellulose as well as the degree of polymerization; thirdly, the elimination of the lignin sheath and redistribution of lignin in the cell wall; and finally, minimizing the particle size and thus enhancing the porosity of the substrate. However, enzymatic hydrolysis encounters some drawbacks in terms of the long time required for the process and the high cost of the needed enzymes. Consequently, research studies have recently been directed towards eliminating these obstacles and reducing these costs [91].

##### Physical Methods

Nanocellulose can be obtained in different sizes by using mechanical extractor homogenizers for industrial application. The main thermodynamic properties improved by using nanocellulose are elastic modulus (Young’s modulus), flexural strength, and tensile strength [92]. Physical methods include electrospinning, self-assembly, phase separation, template synthesis, and drawing.

The physical approach involves the use of mechanical energy or higher energy radiations to generate CNFs. Among the physical techniques, electrospinning is the most widely used for the production of nanofibers owing to the low production cost, efficiency, easy adaptability, and simplicity of the method [93]. The nanofibers produced by electrospinning are characterized by their small diameter, higher surface area, and good porosity. As previously mentioned, an electrospinning apparatus is composed of a high voltage power supplier, a syringe pump, a needle with a blunt tip, and a collector. The process involves the use of a suitable polymer and solvent together with the other needed additives including, e.g., peptides, active agents, nanoparticles, etc. Although this method has been extensively used for the fabrication of nanofibers, the low yield, high voltage usage, and high cost of scaling up are major drawbacks [94].

Self-assembly is another physical method that represents a bottom-up approach where small materials are collected to produce molecular materials like nanofibers. The procedure includes the synthesis of molecules that are needed for self-assembly where the properties of the produced nanofibers are controlled by the interaction between these molecules [95]. Despite the production of thinner and multifunctional nanofibers, this method suffers from complex manufacturing procedures in addition to low production rates [96].

Nanofibers can be also physically fabricated using the phase separation technique. In this method, the selected polymer is allowed to dissolve in a solvent (e.g., Tetrahydrofuran) to produce a homogeneous solution. This is then left to separate into two phases either using a thermal treatment or through adding a non-solvent giving an upper polymer phase and bottom solvent phase, which then results in gelation. Then, the gel undergoes freeze drying, which allows for the easy removal of the solvent [97]. The gelation is the most important stage in the phase separation process as it controls the size and porosity of the polymer [98]. However, this method has its own limitations in terms of using large amounts of polymers and the difficulty of scale-up production in addition to being a time consuming process [99].

Template synthesis is another method used for the production of nanofibers, tubes, or rods. This method is able to produce more flexible fibers with shorter lengths compared to traditional spinning techniques [97]. While unlikely, the major limitation of this method is the need for more physical and chemical processes like dissolution and calcination in order to remove the templates after synthesis [100]. The process of template synthesis comprises three consecutive steps: (a) impregnation to allow coalescence of precursors with templates; (b) formation of solid species during the process; and (c) development of a final product after removing the template. For template removal, different chemical and physical techniques are adopted depending on the nature of the templates [101]. Templates can be generally classified into hard and soft as used in the synthesis process. Hard templates include macroscopic structures and in situ formed templates. Macroscopic structures may involve films and fibers. In situ hard templates are those produced by the in situ physical or chemical conversion of a material in the precursors, and this type includes salt, ice crystals, or carbon. The soft templates are composed of soft matter comprising block copolymers, surfactant, and flexible organic molecules [102,103].

Drawing is considered as a step-by-step process used to generate nanofibers of longer length [80]. In this method, a droplet of the polymer solution used to produce the nanofibers is placed on a silicon dioxide surface, and then a glass rod is allowed to be in contact to this droplet and slowly removed, causing the development of lengthier nanofibers. Subsequently, dry solid nanofibers are produced after evaporating the solvent of the polymeric solution during the drawing process. The selection of the polymer used in the drawing technique depends on its viscoelastic properties, as it should get over the stresses formed during pulling and undergo a higher range of deformations [104]. The limitations of this method are as follows: (i) the selection of the polymer of the ideal viscoelastic properties; (ii) the process is only applicable at a laboratory scale; (iii) the diameter of the produced nanofibers is significantly influenced by the used solvent, where the evaporation of the solvent during drawing results in a subsequent increase in viscosity and the shrinkage of the droplet, thus affecting the formed diameter [105].

##### Chemical Modification and Functionalization

Cellulose is composed of many units called cellobiose that bind from head to tail to form cellulose fiber. Each cellobiose is composed of two glucose units bound by ether linkage between C1 and C4 to form the linear polymer of cellulose. Each unit of the cellulose polymer contains three free hydroxyl groups: one primary group and two secondary groups. Its right end is similar in structure to hemiacetal and is known as the reducing end, while the other end is the non-reducing end [106]. The macromolecule chains of cellulose aggregate to form a fibril with width ranging from 3–5 nm and 100 nm in length. Fibrils join together to form the cellulose fiber with a width reaching 80 nm. The arrangement of these fibers determines the crystal properties of cellulose and affect its physiocochemical properties [32].

Chemical methods are the most commonly used for the production of CNFs with much lower diameter and a higher ratio of length to diameter when compared to those produced by other processes. These procedures facilitate the elimination of the noncellulosic content through alkali treatment or acid hydrolysis [107]. Chemical modifications can be classified into oxidation, esterification, etherification, grafting, and organic/inorganic hybridization.

- Oxidation of Cellulose

Cellulose is a polyhydroxyl-containing compound that makes it susceptible to oxidation by different oxidizing agents. The hydroxyl group at position C6 that is less hindered is the major site for oxidation. The products of oxidation are ketone, aldehyde, and carboxylic acid [108,109]. The ternary oxidation system composed of sodium hypochlorite, sodium bromide, and 2,2,6,6-tetramethyl-1-piperidinyloxy (TEMPO) is an effective system for cellulose oxidation and is widely used for modification of cellulose. Also, other oxidizing agents such as sulfuric acid, persulfuric acid, and hydrogen peroxide can be used [110]. It is clear that the oxidation of the hydroxyl groups at C2 and C3 is more difficult than that at C6, and the drastic conditions needed for the oxidation of these two groups may lead to the complete distraction of the linear polymer. In a highly crystalline polymer, it may be difficult to completely oxidize the C6 hydroxyl group to acid using a large oxidizing agent such as periodate, and instead this will lead to the partial oxidation to aldehyde [111].

In addition to the ability of oxidized cellulose to produce different derivatives of cellulose that can be used in the textile industry, the complete oxidation of carbon 6 in the glucose monomer leads to the formation of a carboxylic acid derivative of cellulose that is considered as one of the most important agents in medical and clinical uses as a highly effective hemostatic agent. Oxidized cellulose-based hemostatic materials have shown local hemostatic and antibacterial activities and appear to be longstanding, commonly used, safe, and efficient hemostats in surgical settings [112]. For hemostatic uses, cellulose is oxidized through the use of nitrogen dioxide gas vapor that produces a material with high carboxyl content [113]. Usually, oxidized cellulose denotes a compatible, absorbable, and biodegradable polymer where the degradation and resorption time in the body can be manipulated by changing the oxidation degree and structure of the original cellulose [114]. More details about the use of oxidized cellulose for surgical applications are provided in Section 5.5.

- Esterification of Cellulose

Another important chemical modification occurring due to the presence of free hydroxyl groups is the esterification reaction. Cellulose can react with both organic acids and their derivatives such as acid chloride or acid anhydride or a mineral acid like sulfuric acid, nitric acid, and phosphoric acid. In addition, cellulose can form esters when reacting with sulfonyl esters, halogenated esters, and carbamates [115]. The main advantage of cellulose esters over cellulose is their solubility and their ability to melt before decomposition; for example, cellulose acetate butyrate exhibits excellent thermal and UV properties, good solubility, and high flexibility [116].

- Etherification of Cellulose

Similar to that with ester formation, cellulose has the ability to totally or partially react with alkylating agents to form ether in the presence of alkalis. To obtain cellulose/ether derivatives, cellulose in the presence of alkali reacts with halogenoalkanes, halogenated carboxylic acids, and epoxides. According to the type of substitution, the ether derivatives can be mixed ether or single ether. Single ether is composed of unsubstituted ether such as ethyl cellulose or methylcellulose. Examples of mixed ether are hydroxyethyl carboxymethyl cellulose, ethyl hydroxyethyl cellulose, hydroxyethyl methyl cellulose, and quaternized hydroxyethyl cellulose [117]. An interesting feature about methylcellulose (single ether) is its reversible thermal gelation behavior. Unlike most polymer solutions that become less viscous with elevations in temperature, the aqueous solutions of methylcellulose become progressively more viscous with rising temperatures and can change into solid matter at about 50 °C. Upon lowering the temperature, the solid-state methylcellulose becomes liquid again [118].

- Grafting of Cellulose

Most of the previously mentioned modifications cause improvements in cellulose properties. However, the increases in molecular weight were not significant and consequently, so were the changes in strength and viscosity and rheological properties of the solution. To solve the above mentioned problem, the insertion of a large group on the cellulose molecule can improve these properties along with the addition of new functionality to the polymer. Grafting is undertaken by the addition of acrylate, methyl acrylate, or methyl methacrylate that lead to the constitution of a new polymer with different characteristics such as water adsorption [119]. There are four main strategies for grafting modification: radical polymerization, free-radical polymerization, ring-opening polymerization (ROP), and ionic polymerization. Radical polymerization is the major procedure used in grafting modifications [119,120].

- Organic/Inorganic Hybridization of Cellulose

Hybridization of cellulose with different organic or inorganic moieties can be considered as a modification of grafting. The different approaches for hybridization include sol–gel process and biomimetic mineralization. Hybridization with inorganic metals such as gold, copper, and silver possesses the benefits of both cellulose flexibility and metal heat resistance [121]. Also, cellulose can be hybridized with metal oxides to improve its antibacterial activity and can then be used for wound dressing [122].

**Figure 3:**
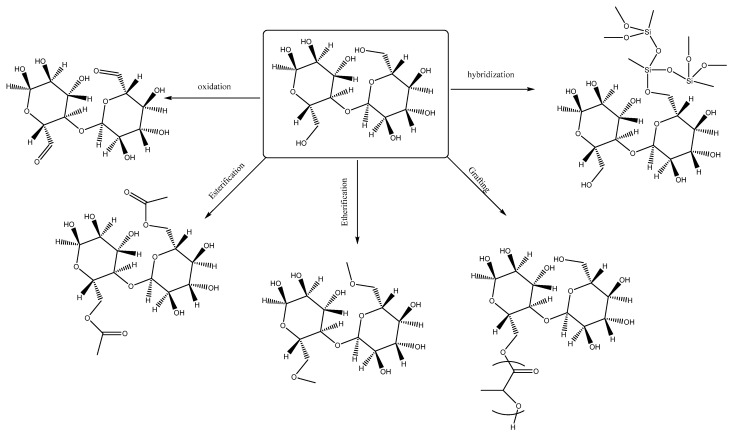
Different methods for the chemical modifications of cellulose.

The different methods for chemical modifications can be summarized as shown in Figure 3.

## 5. Pharmaceutical Applications of Cellulose-Based Textiles

Cellulose is a biocompatible material used for different biomedical applications. As previously mentioned, cellulose is considered as a material of growing interest, stimulating researchers to investigate its potential for biomedical applications as part of promising textiles. Cellulose is characterized by biocompatibility with reduced cytotoxicity, sustainability, and biodegradability [115,123,124] and having tunable physical, chemical, and mechanical properties. Biocompatibility can be defined as the ability to be in contact with a living system without producing an adverse effect [125]. Therefore, by definition, cellulose as a polymer of glucose subunits is considered a biocompatible material, minimizing the risk of adverse reactions while promptly integrating with the human body. As proven by some early reports, cellulose can be considered to be broadly biocompatible and causing no foreign body responses in vivo [25].

Cellulose has a peculiar composition containing both amorphous regions that confer flexibility and plasticity as well as crystalline ones responsible for stiffness and elasticity. This observed balance between amorphicity and crystallinity introduces cellulose as a very interesting biocompatible material [124,126] for different biomedical applications like drug delivery [126], tissue engineering [127], wound healing [128], and antimicrobial [129] and antiviral [130] applications.

As stated above, cellulose is the main component of plant cell walls. It plays an important role in the mechanical strength and shape of the plant [131]. The mechanical strength of a material is determined according to two factors: (a) Young’s modulus, also known as elastic modulus, which measures the stiffness of the material or the resistance to elastic deformation under stress; and (b) tensile strength, which refers to the maximum stress applied to a material that it can withstand while being stretched before breaking. Since plant cell walls bend but do not break, this means that cellulose fibers have high tensile strength and Young’s modulus [125]. Cellulose has a wide range of porosities and mechanical properties depending mainly on the source of production [132]. For instance, cellulose I crystals exhibit extraordinary mechanical properties characterized by their high strength and intrinsic stiffness, thus making them promising candidates for biomedical applications. Interestingly, as they are highly crystalline, CNFs show high specific modulus and strength together with the intermolecular hydrogen bonding between cellulose chains that results in their remarkable intrinsic mechanical properties [133]. Moreover, different types of cellulosic reinforcements have proved very impressive with favorable mechanical properties as seen by their optimum elastic modulus and tensile strength, which make them very convenient for various biomedical applications where stable and strong structures are desired and high mechanical performance is required [133]. Some of the pharmaceutical applications of cellulosic textiles are discussed below.

### 5.1. Tissue Engineering and Regenerative Medicine

Regenerative medicine stands at the forefront of health sciences, offering potential solutions for complex conditions. It harnesses stem cells, tissue engineering (TE), and gene therapy, either individually or combined, to repair, regenerate, or replace damaged cells, tissues, or organs [134,135,136].

Cellulose fibers and nanofibers (CNFs) have garnered significant attention in the field of regenerative medicine due to their distinctive properties and wide-ranging applications. The scientific literature prominently highlights CNFs’ versatility, notably in TE [137] and drug delivery [138].

TE plays a pivotal role in comprehending techniques to regenerate the human body [139]. It combines scaffolds, cells, and biologically active molecules to craft functional tissues, aiming to restore, maintain, or enhance damaged tissues or organs [140]. TE often utilizes both scaffolds [137] and hydrogels [141] as essential components of its approaches.

Scaffolds provide structural support, often mimicking the extracellular matrix and thus aiding in tissue regeneration and repair [142]. Hydrogels, on the other hand, offer a biocompatible and porous environment suitable for cell growth and diffusion [143,144].

In the field of bone TE, scaffolds and hydrogels play a pivotal role [111,145]. A natural bone is characterized by its high porosity with a matrix composed mainly of collagen and hydroxyapatite arranged in a hierarchical structure, and it also contains non-collagenous protein and proteoglycans [22]. The various bone cells, known as osteoblasts, osteoclasts, and osteocytes, are the main factors controlling bone formation and remodeling. Therefore, imitating this complex composition using highly porous biomimetic materials is an essential prerequisite for appropriate bone TE. CNF/hydroxyapatite composites can be used to mimic the natural bone environment regarding biocompatibility and porosity together with the required compressive modulus and compressive strength [146,147].

Cellulose structure can be readily modified to produce a bioactive material with a highly porous nature. This modification can be performed through oxidation with compounds such as TEMPO that yields nanofibers of negative charge and allows the desired dispersion of hydroxyapatite, creating a hydrogel that can be crosslinked [147]. Moreover, CNF can be considered as an excellent carrier of bone morphogenic proteins (BMP) and the vascular endothelial growth factor (VEGF) and thus can be very beneficial in bone tissue engineering. Sukul et al. [148] revealed that the incorporation of BMP and VEGF with CNF resulted in good cell adhesion and proliferation, where BMP enhances osteogenesis and VEGF helps angiogenesis, thus playing an important role in the bone healing process. Additionally, CNF/BMP/VEGF loaded with biphasic calcium showed enhanced proliferation and better cell attachment [149]. In another study, Salama et al. [150] showed calcium phosphate deposition on bones upon using soy protein hydrolysate grafted TOCNFs (TEMPO-oxidized cellulose nanofibril), while treating TOCNFs with SPH also resulted in the precipitation of calcium phosphate and consequently in bone tissue repair.

RCFs offer a multifaceted solution to the challenges of bone TE. They enhance mechanical strength, promote biocompatibility, and create an environment conducive to cell adhesion and growth [151]. In the study conducted by Chakraborty et al., the potential of regenerated cellulose scaffolds as biomaterials for bone TE is highlighted [152]. The researchers used electrospinning to create non-woven nanofibrous scaffolds using cellulose acetate solutions with varying concentrations in an acetone–water system. These scaffolds known as CAS (cellulose acetate scaffolds) have average fiber diameters ranging from 300 to 600 nm. To optimize these platforms, researchers created regenerated cellulose scaffolds (RCS) through deacetylation in alkaline solutions for varying time periods. The RCS underwent heat treatment at different temperatures to explore the benefits of the heating process on mechanical strength enhancement. Surface chemistry, morphology, and physiochemical characterizations were studied using attenuated total reflection Fourier-transform infrared spectroscopy, scanning electron microscopy, and other characterizations. The ideal fabrication conditions were found to be related to solvent system composition, deacetylation time, and heat treatment temperatures. In vitro investigations were conducted on selected RCS samples using MC3T3-E1 osteoblast cells, which showed improved cell adhesion and proliferation. These findings highlight the potential of cellulose-based materials—especially CNFs and RCFs—to improve bone tissue engineering through bettering cell behavior and encouraging bone tissue regeneration.

Skin TE is another field where RCFs are engaged. The skin is the body’s main defense barrier and is composed of dermal and epidermal layers. The epidermis, which makes up the skin’s outer layer, serves as a protective barrier between the outside world and the inner body. The keratinocytes, which make up the epidermal layer, continuously proliferate and differentiate [153]. Quick coverage is needed to help restore the repair and functionality as these are lost due to skin injuries like superficial burns, fissures, or lesions [154]. For complete tissue regeneration, implantable scaffolds have been developed [155,156] in order to create a favorable environment for cells to interact and proliferate. Since commercial solutions are quite expensive, in recent years, a great deal of research has been conducted on nanocellulose-based materials [157,158].

The use of cellulose nanofibers for skin TE applications has several benefits [159]: one, their high surface area that can accommodate various molecules or microbial cells; two, their high-water absorption capacity, which maintains the moisture in the injured skin while absorbing the exudate from the wounds; three, cellulose nanofibers are construction blocks that look like the nanoscale architecture. Several materials were combined with CNF for skin TE purposes. Among those materials, the poly (globalide) (PGl) is created via the enzymatic ring-opening polymerization of unsaturated 16-membered macrolactone globalide. Like most macrolactone polymers, it is non-toxic, highly hydrophobic, semi-crystalline, has a low melting point, and is exposed to hydrolytic or enzymatic breakdowns [160].

Previous studies have demonstrated that PGl can be altered to create useful crosslinked films and fibers. A possible route to successful skin epidermal treatments is the combination of both cellulose and PGl into a bilayer scaffold, where one layer is hydrophobic to prevent water loss from the skin, while the other layer is hydrophilic in direct contact with the skin injury. Layer-by-layer casting at ambient temperatures was used by Amaral and coworkers [161] to create a regenerated cellulose nanofibers (CNF)/PGl bilayer film. The PGl film was first created by pouring 10 mL of a 10% wt/v PGl in chloroform solution onto a Petri plate, which was then allowed to dry at room temperature during the next day. Then, 10 mL of a 0.1% wt/v CNF in water suspension was added to an ultrasonic bath. The suspension was applied to the PGl layer after two hours, and the resulting CNF/PGl film was then allowed to dry at room temperature overnight. For the purpose of removing any remaining solvent and preventing moisture absorption, the produced films were held under vacuum. The top and bottom surfaces of the CNF/PGl film were seeded separately with in vitro spontaneously transformed keratinocytes from histologically normal human skin (HaCaT, AddexBio T0020001) at a density of approximately 50,000 cells/cm^2^. Living/DEAD staining revealed a significant number of living cells on both surfaces of the film, supporting the metabolic activity of the cells. More studies are required to investigate the ability of the CNF/PGl bilayer film to quicken the wound healing process. Based on these results, we may conclude that the CNF/PGl bilayer scaffold has the potential to promote tissue regeneration in skin epidermal treatments by maintaining the viability of live cells. To validate its efficacy in wound healing, in vivo studies and clinical trials ought to be conducted in the future. The scaffold’s ability to repair wounds will be enhanced by improving its design and carrying out further biological tests. Prior to clinical use, safety evaluations and regulatory permissions are also essential processes to guarantee standards compliance and safety.

In addition to scaffolds, hydrogels have also been assuming an increasingly significant role in regenerative medicine applications. In this context, CNFs possess high strength and stiffness [162], low coefficient of thermal expansion [163], high crystallinity [164], hydrophilicity, and an easily modifiable surface [165]. Such excellent properties hold significant potential as foundational components for crafting high-performance and functional hydrogels.

CNFs hydrogels have already demonstrated their utility in different applications: they have been employed in the development of burn dressing, leveraging their properties to aid in wound healing [165]. Additionally, CNFs hydrogels exhibit noteworthy attributes like self-healing capabilities and facilitation of neural regeneration [166]. Impressively, they also exhibit considerable potential for use in bone tissue repair [167] and as a platform for controlled drug delivery, offering both redox and thermo-responsive characteristics [168,169,170]. Together, these results demonstrate how the field of regenerative medicine is developing and how innovative substances like CNFs are essential for increasing therapeutic options.

#### Advances in Cellulose-Based 3D and 4D Printing Technologies for Tissue Engineering

3D and 4D printing have surfaced as smart applications that integrate advanced materials and technologies. 3D printing constructs three-dimensional objects, layer-by-layer, revolutionizing manufacturing and customization [171,172]. Adding a time dimension, 4D printing introduces smart materials that respond to external stimuli, enabling shape-shifting structures [173,174,175].

Regenerated nanocellulose has emerged as a promising material for 3D printing due to its unique properties. The ability to undertake 3D printing of all-regenerated cellulose material (in which both the fibers and the matrix are cellulose [176]) has been explored in a recent study [177], presenting exciting possibilities for creating complex structures.

Further advancements have emerged as other authors [178] introduced 3D-printed composite scaffolds comprised of poly(ε-caprolactone) (PCL) infused with nanocrystalline cellulose and poly (γ-glutamic acid) (PGlu), serving as implants for bone regeneration in rabbit femur bones. The composites, combined with adhered rabbit bone mesenchymal stem cells (MSCs), showed an osteo-regeneration effect. Additionally, a non-invasive 3D printable polymeric hydrogel was devised by incorporating TEMPO-oxidized nanocellulose and carbon dots. This hydrogel, combined with gelatin methacryloyl and polyethylene glycol diacrylate, proved to be ideal for digital light processing bioprinting, boasting high cytocompatibility. With its adjustable structural color and distinctive fluorescent properties, this hydrogel enabled the tracking of human skin cell migration for up to 30 days post-printing [179].

In 2022, Gauss et al. [180] explored the use of regenerated cellulose fibers (lyocell) in additive manufacturing to process poly(lactic acid) (PLA) composites for 3D printing. Composite filaments of PLA reinforced with lyocell fibers were successfully produced for fused deposition modelling. The filaments showed increased Young’s modulus with fiber addition compared to neat PLA and a commercial wood filament, used as a reference, while maintaining substantial porosity. The 10 wt% fiber formulation had the best tensile properties and thermo-mechanical stability compared with the reference and also with other compositions of lyocell/PLA filaments such as those containing 20 wt% of fiber content and 80 wt% of PLA. This approach signifies regenerated cellulose’s potential in enhancing PLA‘s 3D printing properties for diverse applications from customized furniture and construction elements to biomedical and dental solutions. One year later, the same author explored the versatility of lyocell/PLA composites, proving their suitability not just for traditional fused deposition modelling printing but also for innovative 4D printing applications. Researchers used fused deposition modelling to assess the shape-change potential of these composites. The main mechanism involved was the anisotropic swelling of cellulose fibers, which were aligned in the composite during printing [181]. Future studies on the application of these composites in TE will be highly appreciated.

In conclusion, regenerated cellulose has shown remarkable potential in the field of regenerative medicine. Through the integration of intelligent materials and innovative design approaches, 3D and 4D printing have enabled structures that can dynamically change their shapes and properties over time. The combination of cellulose-based polymers and advanced printing techniques has paved the way for bio-derived formulations, ranging from biocomposites to pharmaceuticals, with diverse applications. As ongoing research and development continue to refine these processes, the synergy between regenerated cellulose and smart printing applications holds the promise of transformative advancements across various biomedical industries [182,183].

**Figure 4:**
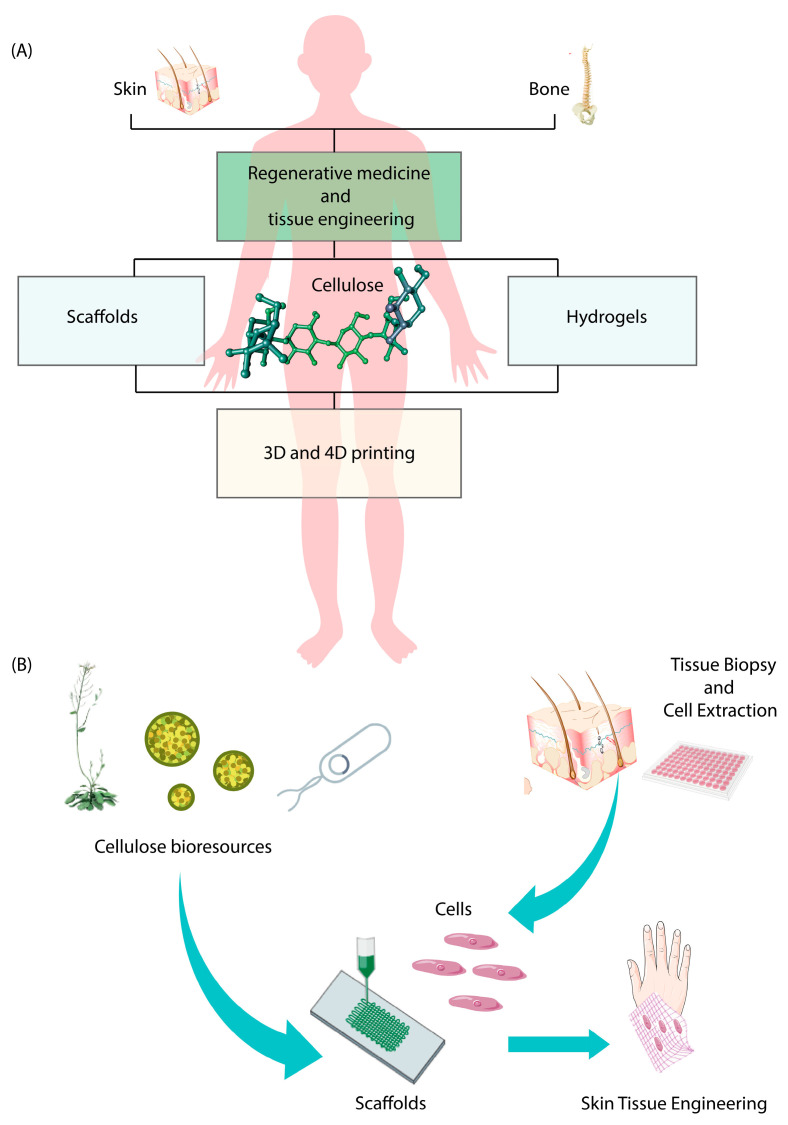
(**A**) Cellulose fibers are versatile and useful materials in the realm of regenerative medicine and tissue engineering. Cellulose fibers are suitable for both scaffold and hydrogel crafting via 3D and 4D printing. The latter allows cellulose to obtain peculiar properties such as stimuli-responsive behavior. These technologies pave the way for new advanced strategies for skin and bone tissue regeneration. (**B**) Cellulose can be obtained by different sources to then be regenerated and used to make scaffolds. Those devices are suitable for cell growth and differentiation making them a promising tool for regenerative medicine in, for example, skin tissue engineering (Created with bioicons.com).

Figure 4 highlights the use of cellulose fibers in the regenerative medicine field.

### 5.2. Antimicrobial Uses

The strategic integration of antimicrobial agents into cellulosic and nanocellulosic pharmaceutical textiles marks a pivotal advancement in the ongoing battle against microbial threats within healthcare and beyond. This innovative approach capitalizes on a wide spectrum of antimicrobial agents, each harnessing distinct mechanisms to counteract the proliferation of microorganisms. These agents span a rich diversity, encompassing metal nanoparticles [184], natural extracts originating from plants [185], and engineered synthetic compounds [28]. The judicious selection and incorporation of these agents empower the creation of textiles with multifaceted defenses that effectively mitigate the presence of pathogens on their surfaces [24].

Metal nanoparticles, particularly silver (Ag) and copper (Cu), have garnered substantial attention for their antimicrobial properties. Metal nanoparticles can be composed of different metals, with silver nanoparticles (AgNPs) being of particular interest [186]. Ag and its salts have a long history of medical use, with metallic silver being utilized since ancient times to heal wounds and aid in food preservation. Silver salts, especially in the form of creams or ointments, were extensively employed as antiseptics for treating burns and wounds during World War I [186]. AgNPs possess strong antimicrobial properties due to their large surface area that enhances their contact with microorganisms. They attach to cell membranes, permeate bacteria, and interact with sulfur-rich proteins and phosphorus-containing DNA within the cells. By targeting the respiratory chain and cell division, these nanoparticles lead to bacterial death. The release of silver ions further boosts their bactericidal potency [186]. Integrating these nanoparticles into textiles capitalizes on their ability to interact with bacterial cell membranes.

In parallel, the integration of natural extracts derived from plants, or insects, into cellulosic textiles offers an eco-friendly approach to combat microbial growth. Plants synthesize a plethora of secondary metabolites, such as tannins, terpenoids, alkaloids, and flavonoids, and these compounds possess inherent antimicrobial attributes developed as part of their evolutionary defense mechanisms. These compounds can target various microbial components interfering with vital cellular processes. For instance, they might hinder microbial enzyme activity, disrupt DNA replication, or interfere with nutrient uptake, collectively impeding the ability of microorganisms to thrive. Recently, some authors combined the antimicrobial properties of metallic NPs with those of natural substances, obtaining composite materials with enhanced antimicrobial effects and therefore, potentially usable in the biomedical field [187].

Synthetic antimicrobial compounds (like sulfadiazine, tetracycline, etc.), introduce a chemically engineered dimension to antimicrobial formulation. These compounds are designed to interact with microbial cell surfaces, leading to the disruption of the integrity of the cell membrane. Moreover, they can affect intracellular processes, interfering with crucial metabolic pathways and rendering microorganisms incapable of sustaining growth. The incorporation of synthetic compounds complements natural antimicrobial agents, presenting a synergistic approach that amplifies the overall antimicrobial efficacy [188].

Studies showing the merging between cellulose or nanocellulose and the active compounds mentioned above are discussed in detail, aiming to highlight the most recent innovations in the field.

#### 5.2.1. Use of Metal Nanoparticles

Due to the attributes mentioned earlier, composite threads have been formulated in recent years, embedding metal nanoparticles within the fabric structure. The combination of metal nanoparticles with biocompatible biopolymers, such as cellulosic matrices, have the significant advantage of mitigating potential harm to eukaryotic cells [189].

Cellulose can be utilized either in its initial form or in the form of nanocellulose. As emphasized by Zhang [190], because of the significant size difference between cellulose fibers and AgNPs, it is essential to achieve a more uniform and higher ratio of Ag content in composites for biomedical applications, and this can be accomplished using nanocellulose. In their study, Zhang developed a hydrothermal method for synthesizing AgNPs on nanocellulose in aqueous solutions, with nanocellulose serving as both the reducing agent and stabilizer.

Recent developments in this field revolve around environmentally friendly strategies to produce nanocellulose-metal antimicrobial composites. These methods incorporate green reducing agents, such as ascorbic acid, into the fabrication of nanocellulose-AgNPs [191].

Additionally, copper oxide (CuO) and zinc oxide (ZnO) nanoparticles can be synthesized in situ through ion reduction [192] or via precipitation, followed by subsequent coating onto cellulose [193,194]. The antimicrobial capabilities of these composite materials have been evaluated in various ways. Table 1 presents an overview of the studies illustrating the preparation of cellulose-metal NPs for antimicrobial purposes.

#### 5.2.2. Use of Natural Products

Plant-derived bioactive compounds, referred to as phytochemicals, serve as valuable resources for medicinal purposes [205]. These compounds possess a wide array of antimicrobial attributes shaped by their chemical characteristics, origins, and quantities. Comprising a complex mixture of molecules, their collective actions can amplify their efficacy against microbes [206].

These compounds chiefly target microbial cells by compromising the integrity and functionality of the plasmatic membrane. They can also impede efflux pumps, disrupting cell communication in multidrug-resistant pathogens. Certain compounds modulate protein interactions, mitosis, apoptosis, and intermediary metabolism while inducing cytoplasmic coagulation and deterring biofilm formation, which is a protective mechanism for pathogens. Additionally, plant extracts contain multiple antiviral components that intervene at various stages of viral replication [207].

Bioactive plant compounds can be classified into various groups based on their chemical structures, composition, biosynthetic pathways, or solubility. These groups encompass alkaloids, phenolic compounds, sulfur-containing compounds (polysulphides), coumarins, and terpenes [187].

Natural alkaloids exhibit antibacterial effects by damaging bacterial cell membranes, altering DNA functions, and inhibiting protein synthesis [208]. Phenolic compounds act on bacterial cells by impairing the bacterial membrane, inhibiting enzymes and toxins, and suppressing bacterial biofilm formation [209]. The precise mechanism of action for polysulphides remains unclear; among the various hypotheses, thiolation reactions or hydrophobic interactions are possibilities [210]. The antibacterial activity of coumarins is correlated with the polarity of oxygen-containing substituents on the benzene ring [211]. Terpenes found in essential oils have the capacity to modify the cell envelope and cytoplasmic stability, leading to cellular damage [212].

To facilitate the biomedical applications of these compounds, they can be incorporated into natural polymers such as chitosan, cellulose fibers, or nanocellulose. In recent years, numerous studies have focused on optimizing the synthesis conditions of these intricate biomaterials. The literature presents several studies for the use of natural plant products in combination with cellulose or nanocellulose for antimicrobial purposes. Since the extraction conditions of natural products influence their activity, several studies have included information related to the solvents utilized in extraction procedures. The significance of this aspect has been notably highlighted in a recent study [27], where the extraction of compounds from plants was carried out in water or glycolic alcohol. The obtained results revealed the absence of antimicrobial activity, which is likely attributable to the extraction method not providing a sufficient concentration of active principles. Another study highlighted the use of basil ethanolic extract as an antibacterial agent in nanocellulose. A slight inhibition of *S. aureus* growth was detected in a nanocellulose sponge loaded with basil ethanolic extract [213].

Among other natural compounds derived from insects and loaded onto cellulose fibers, propolis has garnered significant interest. This product was used to prepare various formulations for biomedical applications. In one study, ethanol was employed to produce propolis extract, which was then incorporated into CNF/poly(vinyl alcohol) matrices. The resulting hydrogels exhibited substantial antimicrobial effects against *E. coli*, *S typhimurium*, *S mutans*, and *C albicans*, which are attributable to the high content of phenolic acids and flavonoids in propolis [214].

#### 5.2.3. Use of Synthetic Compounds

In recent advancements within the field of antimicrobial materials, synthetic compounds with potent antimicrobial properties have gained considerable attention. These compounds, carefully engineered for their antimicrobial efficacy, are now being harnessed in combination with cellulose fibers to create innovative and highly effective biomedical materials. This integration not only leverages the inherent biocompatibility of cellulose but also capitalizes on the versatile properties of synthetic compounds to combat microbial threats. In this context, the conjugation of synthetic antimicrobial compounds with cellulose fibers opens up promising avenues for the development of advanced materials with applications in wound care, medical textiles, and various other biomedicine-related fields, as demonstrated by recent publications. In a recent manuscript, the authors evaluated the antimicrobial activity of CNF sheets incorporated with ceftriaxone, which is an antibiotic belonging to the cephalosporin group. Various percentages (0%, 5%, 10%, 15%, and 20% based on polymer weight) of ceftriaxone were integrated with a polymer, like polyvinyl alcohol, using the electrospinning technique to produce nanofiber sheets. The results indicated that the nanofiber sheets containing ceftriaxone exhibited potential inhibitory activity against *E. coli* and *S. aureus* [28].

### 5.3. Antiviral Studies

The applications of cellulose in defense against microorganisms are not limited to bacteria and fungi. There are also studies regarding viruses, although these are not as numerous. This section will provide some examples of the various applications of cellulose in antiviral preparations. In fact, in this field, cellulose fibers can be used both as a vehicle for therapeutic agents and as a material to control the spread of viruses (innovative antiviral textiles).

In a recent study conducted by Qian et al. [130], an innovative method has been proposed to prepare cotton fabric with persistent antibacterial and antiviral properties over time. To summarize, the authors introduced a technique capable of creating antimicrobial cotton textiles by integrating Cu ions into the cotton structure at the molecular level. This process hinges on disrupting the hydrogen bonds between cellulose chains, allowing Cu(II) ions to permeate swollen cellulose materials and establish a stable Cu(II) ion-cellulose complex. This method is realized through a one-pot reaction where cotton fabric is immersed in a Cu(II)-saturated NaOH solution, ensuring that the resulting Cu(II) ion-textile (Cu-IT) remains highly resistant to exposure to air, water, and physical wear and tear. Cu-IT exhibits effective interactions with viral genomes, thereby impeding virus replication, and it efficiently eliminated bacteria and fungi by damaging cell membranes and inducing the production of reactive oxygen species.

**Figure 5:**
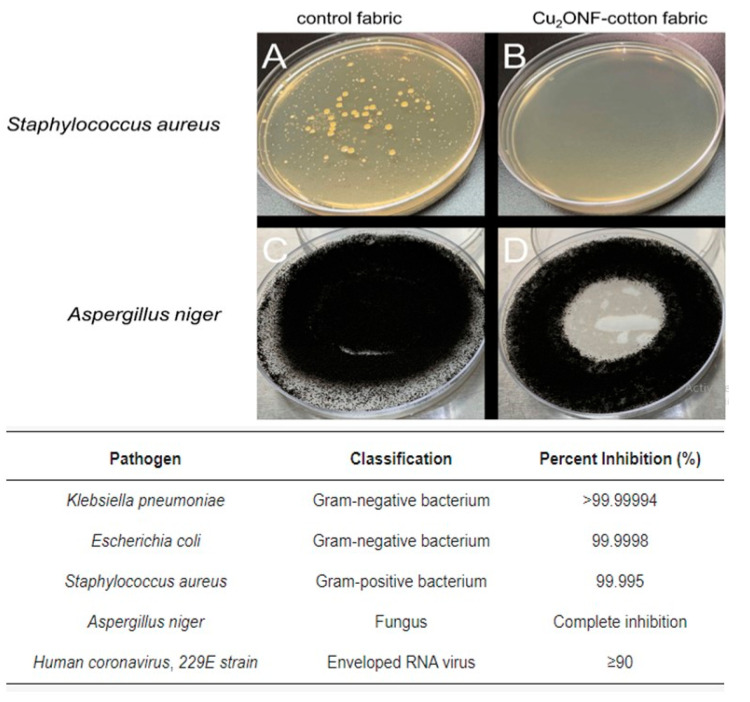
Cu_2_O nanoparticles in cotton fabrics demonstrated excellent antibacterial, antifungal, and antiviral activities. Antibacterial inhibition activity for (**A**) control fabric and (**B**) Cu_2_O NF cotton fabrics. Antifungal inhibition for (**C**) control fabric and (**D**) Cu_2_O NF cotton fabrics [215]. Abbreviations: NF; Nanoflower.

Similar findings were achieved in another study, where nanoflower-shaped Cu(I) oxide (Cu_2_O) NPs were fabricated in-situ within cotton fabric under gentle conditions, devoid of supplementary chemical reducing agents, and derived from a Cu(II) precursor. The Cu_2_O securely embedded within the cotton textile and demonstrated persistent antibacterial effectiveness (≥99.995%) against *K. pneumoniae*, *E. coli*, and *S. aureus*, total antifungal efficacy (100%) against *A. niger*, and noteworthy antiviral performance (≥90%) against human *coronavirus*, strain 229E, even following 50 washing cycles (Figure 5) [215].

It is noteworthy that at present, no single formulation or preparation method has been conclusively identified as superior to another. Extensive literature data suggests that ongoing research is dedicated to the exploration and identification of formulations that effectively enhance the antimicrobial properties of various active ingredients. The focus remains on optimizing formulations not only to maximize antimicrobial efficacy but also to improve patient compliance. Despite the absence of a definitive conclusion regarding the superiority of one formulation over another, the collective efforts in research aim to uncover formulations that not only excel in antimicrobial activity but also contribute to enhanced patient adherence and overall treatment outcomes.

### 5.4. Wound Dressing

The skin is the largest organ of the body and is the most vulnerable and susceptible to injuries, thus affecting its function as the main barrier against the external environment [216]. Skin wounds can be classified as acute or chronic: the former happens incidentally and healing takes place in 8 to 12 weeks, while the latter happens over time, such as in diabetic foot ulcers where the healing of such wounds is more challenging and the time required for complete healing cannot be determined precisely [217]. After skin damage, the process of wound healing is a complicated yet very coordinated biological process including hemostasis, inflammation, cell proliferation, and maturation, involving different types of cells, growth factors, and extracellular matrix components (ECM) [218,219]. Thus, the main purpose of a wound dressing is to maintain the hydration of the injured site, protect it from being exposed, absorb/remove excess exudates, enhance the healing process, and keep the wound safe and away from external contaminants [220].

The most commonly used wound dressings include antibacterial or antibiotic-loaded creams, hydrogels, or ointments. However, these topical preparations require frequent cleaning and reapplication [221]. Therefore, the use of nanofibers-based wound dressings has been extensively growing owing to their biodegradability, high air permeability, hypoallergenic properties, high surface-to-volume ratio, and their ability to absorb secretions from the injured site in addition to their effective enhancement of cell proliferation, allowing for the gradual release of nanofibers-loaded active agents into wounded areas [18,222,223].

Wound dressings are produced using numerous fabrication techniques—most commonly solvent casting, electrospinning, electrospraying, and 3D printing. The selected fabrication technique should fulfill the main criteria for the fabrication of an effective dressing of high porosity in order to guarantee wound respiration and to help the permeability of oxygen gas [224]. Solvent casting is considered the most commonly utilized technique producing flexible, easy-to-apply wound dressing films with enhanced gas permeability while preventing bacteria/liquid permeation [225]. However, some difficulties are encountered with the production of porous films, as the method lacks adequate control over the porosity as well as the permeability of the produced dressings in addition to the need for salt or particulate leaching [226]. On the contrary, the electrospinning technique has been extensively used for the production of wound dressings. This method provides dressings with enhanced properties suitable for various functions with controlled porosity and a high surface-to-volume ratio [227]. Similarly, the electrospraying technique has been widely explored for the preparation of various wound dressings [228]. Interestingly, of all the methods used for fabricating wound dressing nanofibers, 3D printing attracts the most attention. This technique guarantees excellent control over porosity and can be loaded with various biomaterials and/or active therapeutic agents necessary for different stages of wound healing. This unique property is offered only by the 3D printing technique: producing several layers of biomaterials having different compositions arranged over each other to obtain finally arbitrary geometries [229,230].

**Figure 6:**
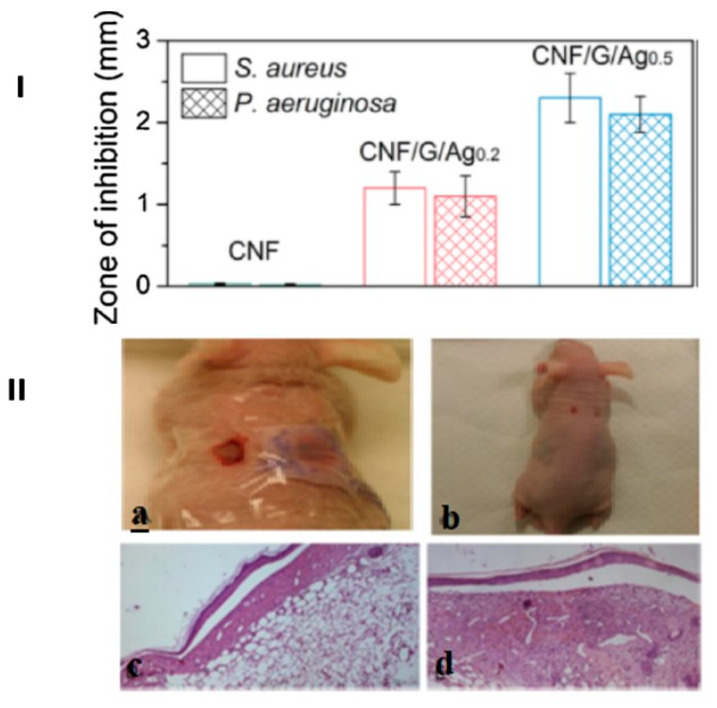
(**I**) Zones of inhibition of silver nanoparticles and gelatin loaded CNF wound dressing compared to plain CNF dressing against *S. aureus* and *P. aeruginosa*. Reprinted from reference [232], with permission from Elsevier. (**II**) Full thickness induced wound demonstrating the effect of NFC wound dressing. (**a**) Covering of treated wounds with NFC dressing (on the right) while the control (on the left) was left untreated. (**b**) The NFC wound dressing separated itself after 8–9 days. (**c**,**d**) Photomicrographs of the control and injury treated with NFC dressing after being stained with hematoxylin and eosine [235].

Accordingly, CNFs are considered as ideal substrates for wound dressings owing to their highly porous structure, excellent biocompatibility, and good mechanical properties in addition to their ability to act as a barrier against any external factor, thereby preventing secondary infections. CNFs provide various chemical reaction sites for hydroxyl groups with the potential to accommodate glycosides, proteins, polysaccharides, nanoparticles, antibiotics, local anesthetics, or other active agents at the injured area [231]. Several studies have demonstrated the superior wound healing potential of CNF dressings. Aminated silver nanoparticles and gelatin loaded CNF was prepared by Liu et al. [232] and showed adequate mechanical characteristics with pronounced antibacterial properties maintaining the homeostatic conditions and proper fluid balance at the wound site (Figure 6I). Both the in vitro and in vivo evaluations highlighted the good biocompatibility, distinguished efficacy, and wound healing capabilities of the dressing. In another study, Md Abu et al. [233] prepared CNFs loaded with honey using polyvinylpyrrolidone as a binder, and they showed pronounced wound healing properties owing to their anti-microbial efficiency against both Gram-negative and Gram-positive bacteria. Similarly, calcium ion cross-linked CNFs dressings have been proven to have good biocompatibility and convenient maintenance of moisture with good mechanical stability, where calcium plays an additional role in homeostasis and epidermal cell migration and regeneration [234].

Interestingly, functionalized CNFs dressings have also been investigated in clinical trials to help in skin healing and regeneration for burn victims. Compared to the commercial products existing in the market, such as Suprathel R, cellulose dressings have outstanding features. Despite the dressing not being antibacterial, it prevented any bacterial growth and second infection. The mechanical and physical properties of the dressings can be readily tailored to suit a patient’s requirements. Upon application on the wound, the dressing can be easily attached and then detaches smoothly on its own following the complete regeneration of the skin (Figure 6II) [235].

### 5.5. Surgical Uses

In the preceding paragraphs, the extensive discussion was focused on cellulose applications in the tissue regeneration field as antimicrobial or antiviral preparations or as wound dressings. However, cellulose fibers also play a crucial role in the surgical field due to their hemostatic activity [236]. Specifically, oxidized cellulose fibers are utilized for this purpose [112]. As of today, the hemostatic mechanism of textiles based on oxidized cellulose remains not fully understood. Presumably, a physical process is involved. When these textiles are applied to a bleeding site, they extract water from the blood and entrap blood corpuscular elements (platelets, red blood cells, and other active components), thereby increasing the concentration of coagulation factors [237].

Currently, various products with hemostatic activity, comprising oxidized cellulose, are available in the market. These include woven or nonwoven fabrics [238], films [239], powders [240], gauzes, or multilayer filaments with excellent hemostatic results [241]. Presently in clinical applications, the most commonly used materials based on oxidized cellulose are woven or nonwoven fabrics [242].

Moreover, tissue adhesives and sealants are becoming increasingly popular for wound closure and healing applications, particularly when traditional approaches, such as sutures, are ineffective as they are of high cost, can cause discomfort in handling, and are prone to infections. Tissue adhesives and sealants have shown great efficiency in avoiding blood loss that might occur as a result of major injuries or during surgeries [243]. Although there are various commercially available bioadhesives, none of them are suitable for the reparation of elastic and soft tissues. The crucial trade-off is between mechanical characteristics and biocompatibility. Another disadvantage of most commercially available products is their poor performance in moist and highly dynamic media in the presence of blood [244,245].Cellulose fibers can be incorporated in composite bioadhesive matrices to achieve high mechanical strength combined with biocompatibility and other desirable properties. This integration can increase cohesiveness by reinforcement of the polymeric matrix. Fiber-reinforced polymers have many potential uses in medical applications [246].

Cellulosic fibers were integrated into a gelatin/alginate hydrogel and resulted in a composite bioadhesive matrix with high mechanical and physical properties [247]. It was concluded that the fibers’ geometry had a significant effect on the mechanical properties of the preparation. Fibers with a high aspect ratio (ratio between length and diameter) have been shown to be more effective for reinforcement of materials compared to those with a low aspect ratio [248].

## 6. Regulatory and Safety Considerations Associated with the Use of Cellulose Textiles in Pharmaceutical Products

The pharmaceutical industry operates under stringent regulatory standards to ensure the safety and efficacy of medicinal products. As previously mentioned, cellulose textiles are commonly utilized in various pharmaceutical applications, necessitating meticulous attention to regulatory and safety considerations to meet compliance requirements.

The principal Regulatory Standards are as follows.

- US Pharmacopeia (USP): The USP provides monographs and guidelines for various pharmaceutical ingredients and excipients. Cellulose textiles used in pharmaceutical applications must adhere to USP standards [238].
- European Pharmacopoeia (Ph. Eur.): For pharmaceutical products intended for the European market, compliance with Ph. Eur. requirements is essential. The Ph. Eur. includes specific monographs on materials like cellulose and related substances [249].
- FDA Regulations: In the United States, the Food and Drug Administration (FDA) sets strict regulations for materials used in pharmaceutical products. Cellulose textiles must comply with these regulations to ensure product safety.

Regarding the safety considerations, the following should be addressed.

- Biocompatibility: Cellulose textiles should be evaluated for biocompatibility to ensure that they do not induce adverse reactions when they come into contact with pharmaceutical products or ultimately, with patients [250].
- Compatibility with other ingredients: Cellulose textiles must not adversely affect the stability, efficacy, or quality of pharmaceutical formulations, and compatibility studies are essential [251].
- Extractable and leachable: Cellulose textiles can release substances that may interact with pharmaceutical formulations. Analyzing them is crucial to identify any potential risks [252].
- Particle contamination: Contamination by cellulose particles, such as fibers or fines, should be minimized to prevent impurities in pharmaceutical products [251].
- Sterility: For certain pharmaceutical applications, such as wound dressings or surgical products, ensuring the sterility of cellulose textiles is of paramount importance [253].

## 7. Challenges and Future Directions

As explained above, cellulose is a reliable and abundant polymer with renewable sources. The latest developments in the cellulose-based textile industry involve the use of more sustainable processes for a fast and highly demanding pharmaceutical market.

Despite the merits of using cellulose in the manufacture of pharmaceutical and medical textiles like its biocompatibility, moisture absorbability, and sustainability, some limitations and challenges may hinder its development.

One of the main challenges facing the production and commercialization of cellulose is the methods of production. As it is from a natural source, the diversity of the structures of raw materials, which are primarily species-dependent, represents a major obstacle in terms of creating a unique method for cellulose fibers production. Another limitation is that as it is a natural polymer, cellulosic textiles may suffer from short lifespans due to lower durability compared to synthetic textiles. Moreover, the extraction and chemical treatment methods using different solvents can have several environmental implications if not well-managed. Ameliorating the fabrication techniques and application of new protocols are demanding prerequisites in order to maintain the consistency of the fiber properties from one batch to another as well as reducing the needed chemicals, equipment, and time, thus minimizing rejections of the final product and decreasing the production costs and final price. Trials to decrease costs are directed towards using non-wood species for production or further developing already existing functionalization techniques to increase the charge density of fibers. At the same time, it is also necessary to find new approaches to overcome the risk of clustering and aggregation within fiber walls during drying, consider the clogging problems of the equipment, and use less aggressive chemicals and more straightforward green strategies.

Although enhanced moisture absorbance is a unique property of cellulosic textiles, in some cases, it may not be favored when quick drying is required.

Furthermore, the mechanical properties of cellulose fibers may not always meet the specific requirements of pharmaceutical textiles. For example, they may lack the desired strength, flexibility, or moisture absorption properties needed for certain applications. Developing cellulose fibers with tailored properties to meet these requirements can be a challenge.

Moreover, regulatory considerations and standards can pose additional challenges for meeting the necessary regulatory requirements for safety, efficacy, and quality control.

While we have made significant progress in the area of cellulosic textiles, it is important to acknowledge that the journey of developing this field is far from complete. Scientists should continually find ways to address the limitations and improve the outcomes of using cellulosic textiles. Addressing these challenges requires ongoing research, development, and collaboration between factories concerned with cellulose preparation, textile manufacturers, pharmaceutical companies, and regulatory bodies to ensure the successful integration of cellulose fibers in pharmaceutical textiles. Finally, exploring new applications of cellulose fibers is urgently required for making their production and commercialization industrially worthwhile.

## Figures and Tables

**Table 1:** An overview on the combination of metal nanoparticles with cellulose for antimicrobial applications.

Nanoparticles Used	Pathogens Combated	Final Product	Key Findings	Ref.
AgNPs	*Pseudomonas aeruginosa*	Topical hydrogel composed of nanocellulose and hyaluronic acid and loaded with AgNPs.	Bactericidal efficacy of 99.99%.	[129]
AgNPs	*E.coli*, *S. aureus*, *P. aeruginosa.*	Microfibrillated cellulose/silver nanocomposites hydrogels incorporated in polyvinyl alcohol hydrogel.	Significant antimicrobial activity.	[195]
AgNPs	*E. coli*, *S. aureus*, *P. aeruginosa two clinical isolates multidrug resistant S. aureus* and *P. aeruginosa.*	Orange peel cellulose bio-composites loaded with AgNPs	Excellent antimicrobial activity against both Gram-positive and Gram-negative strains.	[191]
AgNPs	*S. aureus*; *E. coli*; *Vibrio cholera*, *P. aeruginosa*, *Bacillus subtilis*; *Enterococcus faecalis*; *Eubacteriumlentum*; *Staphylococcus epidhermis*; *Trichophytonrubru*, *Candida albicans*, *Aspergillus flaves*, *Trichophytonsimi*, *Trichophytonmentagrophytes*	Regenerated cellulose matrix (leaf extract of Cissampelos pareira) loaded with AgNPs.	Antibacterial and antifungal properties were enhanced with increasing AgNO_3_ concentration.	[196]
ZnO and Cu NPs	*E. coli*	Cotton fibers loaded with ZnO and CuNPs.	The cotton fibers were effective against *E. coli* under photoirradiation.	[197]
AgNPs	*S. aureus*, *E. coli* *C. albicans*	Composite membranes based on Graphene oxide/hydroxyapatite/cellulose fibers containing AgNPs.	Strong antimicrobial activity.	[198]
AgNPs	*S. aureus*, *E. coli*	AgNPs-coated cellulose sheets	AgNs-coated cellulose sheet showed good antimicrobial properties against *S. aureus*: 54.5% and *E. coli*: 43.8%.	[199]
Titanium dioxide (TiO_2_NPs)	*S. aureus*, *E. coli*	Cotton fibers loaded with curcumin/TiO_2_ nanocomposite.	Results indicated that *E coli* had a higher response than *S. aureus*, which could be due to variations in bacterial cell wall organization structure.	[200]
CuNPs	*S. aureus P. aeruginosa* bac	Cotton fibers extracted from *A. aspera* and loaded with CuNPs.	Good antibacterial activity.	[201]
AgNPs	*B. subtilis*, *S. aureus*, *M. luteus*, *P. aeruginosa*, *E. coli*, *E. aerogenes.*	Cotton fibers extracted from *Cassia roxburghii* and loaded with AgNPs.	High antibacterial activity.	[202]
Cu_2_ONPs	*E. coli*, *S. aureus*, *A. brasiliensis*	Cellule textiles loaded with Cu_2_ONPs	All fabrics treated with Cu_2_ONPs exhibited a 100% inhibitory effect against *E. coli* and *S. aureus*. As for the antifungal effect against *A. brasiliensis*, Cu_2_ONPs-treated fabrics demonstrated notable antifungal activity.	[203]
AgNPs, ZnONPs and TiO_2_NPs	*S. aureus*, *S. epidermidis*	Cotton nanofibers (derived from *Allium cepa*) loaded with Ag, ZnO and TiO_2_ nanoparticles	Results demonstrated that bacterial growth of both *S. aureus* and *S. epidermidis* were diminished after treatment with cotton coated with silver or titanium NPs.	[204]

## Data Availability

Data sharing is not applicable to this article as no new data were created or analyzed in this study.
